# Greywater Disposal Practices in Northern Botswana—The Silent Spring?

**DOI:** 10.3390/ijerph121114529

**Published:** 2015-11-13

**Authors:** Kathleen A. Alexander, Adil Godrej

**Affiliations:** 1 Fisheries and Wildlife Conservation, Virginia Tech, Blacksburg, VA 24061, USA; 2 Center for African Resources: Animals, Communities and Land Use, Kasane, Botswana, South Africa; 3 Occoquan Watershed Monitoring Laboratory, Virginia Tech, Manassas, WV 20110, USA; E-Mail: agodrej@vt.edu

**Keywords:** sanitation, public health, greywater, pit latrine, ground water contamination, Botswana, pollution, health behavior

## Abstract

Disposal of greywater is a neglected challenge facing rapidly growing human populations. Here, we define greywater as wastewater that originates from household activities (e.g., washing dishes, bathing, and laundry) but excludes inputs from the toilet. Pollutants in greywater can include both chemical and biological contaminates that can significantly impact human, animal, and environmental health under certain conditions. We evaluate greywater disposal practices in nonsewered, low-income residential areas in Kasane (264 dwellings/ha), Kazungula (100 du/ha), and Lesoma (99 du/ha) villages in Northern Botswana through household surveys (*n* = 30 per village). Traditional pit latrines were the dominant form of sanitation (69%, *n* = 90, 95% CI, 59%–79%) while 14% of households did not have access to onsite sanitation (95% CI 0%–22%). While greywater disposal practices varied across villages, respondents in all sites reported dumping greywater into the pit latrine. Frequency varied significantly across villages with the highest level reported in Kasane, where residential density was greatest (*p* < 0.014, χ^2^ = 9.13, 61% (*n* = 23, 95% CI 41%–81%), Kazungula 41% (*n* = 22, 95% CI 20%–62%), Lesoma 13% (95% CI 0%–29%). Disposal of greywater in this manner was reported to limit contamination of the household compound and reduce odors, as well as pit latrine fecal levels. Some respondents reported being directed by local health authorities to dispose of greywater in this manner. Environmentally hazardous chemicals were also dumped directly into the pit latrine to reduce odors. With high household to pit latrine ratios particularly in rental properties (4.2 households, SD = 3.32, range = 15 units, average household size 5.3, SD = 4.4), these greywater and pit latrine management approaches can significantly alter hydraulic loading and leaching of chemicals, microorganisms, and parasites. This can dramatically expand the environmental footprint of pit latrines and greywater, increasing pollution of soil, ground, and surface water resources. Challenges in greywater disposal and pit latrines must be addressed with urgency as health behaviors directed at minimizing negative aspects may amplify the environmental impacts of both greywater and pit latrine excreta.

## 1. Introduction

The most alarming of all man’s assaults upon the environment is the contamination of air, earth, rivers, and sea with dangerous and even lethal materials. This pollution is for the most part irrecoverable; the chain of evil it initiates not only in the world that must support life but in living tissues is for the most part irreversible.

—Rachel Carson, Silent Spring [1]

Human waste is identified as a critical threat to human and animal health as well as ecosystem function. Addressing escalating sanitation needs is a significant challenge facing growing urban areas worldwide, a particularly critical issue in impoverished regions. The situation is predicted to only become worse with population growth over the next 30 years projected to occur dominantly in urban centers, particularly in Asia and Africa, creating extraordinary pressure on already inadequate infrastructure [2,3].

Disposal of greywater is an increasing environmental challenge in congested urban areas and has largely been neglected with development focus directed at improving excreta disposal and provision of clean water [4,5]. Greywater is defined as wastewater that originates from household activities, such as washing dishes, bathing, and laundry, but does not include inputs from toilets [6,7,8]. Previous studies on greywater in South Africa have found high levels of chemical and detergent pollutants as well as fecal bacteria that exceeded 1800 colony forming units (cfu) per 100 mL [9,10]. While there is evidence to suggest that fecal indicator bacteria may over-estimate fecal loads [11], infectious disease causing microorganisms (e.g., viral, bacterial, and protozoal) can occur in this medium [12]. The risk will be influenced by the health status and age of the members of the household from which the greywater emanates, type of housing area (formal or informal), level of services available, household income, and local environmental conditions among other factors [13,14,15]. Chemical pollutants in greywater can vary considerably by area as well, depending on product availability in the local markets and use practices of a particular household [7]. Greywater can also have direct impacts to the environment through erosion, pooling, scum, and grease build up [16]. The nature of the impacts will be influenced by factors such as soil surface properties, topography, water table depth, and proximity of the area to sensitive environments [17].

Where water access is improved in the absence of greywater management, environmental pollution and public health impacts can increase dramatically [10]. Greywater production rates are estimated to increase two- to three-fold in households where reticulated water access is developed within the yard ((e.g., stand pipe), reviewed [10,18]). Per person, greywater production can be significant [19] but can vary considerably, depending on local conditions. In non-sewered regions in both urban and rural environments where dense residential dwellings occur, greywater presents an insidious health challenge that can reach beyond the household to contaminate limited water resources—impacting sensitive environments.

As with other types of human waste, many domestic and wild animals species will utilize greywater resources if available, increasing the potential for pathogen spillover from humans to animals and chemical exposures. This is particularly important in dryland regions, such as Botswana, where greywater may attract water-dependent animals in an environment that has limited surface water availability. Animal consumption of greywater that is contaminated with human associated bacteria may also facilitate the movement of antibiotic resistant microbes from humans to animals, potentially contributing to the environmental spread of antibiotic resistance. For example, banded mongooses (*Mungos mungo*) in the same area in which this study was conducted regularly utilize greywater resources from local hotels and residences. High levels of multidrug resistance were found among fecal isolates of *Escherichia coli* collected from this species [20,21]. When comparing mongoose and human fecal *E. coli* isolates, similar antibiotic resistance profiles were found together with a high degree of genetic similarity, suggesting that fecal microbial movement is occurring regularly between humans and mongoose [21]. Further study is needed to fully evaluate how greywater consumption by domestic and wild animals may influence the movement of microorganisms and antibiotic resistance across hosts and landscapes, particularly in more arid regions where surface water is limited.

Greywater can be a critical source of environmental pollution that will only increase as the world population grows and sanitation infrastructure lags behind. Here, we evaluate greywater disposal practices and sanitation among households in non-sewered, low-income areas in Kasane, Kazungula, and Lesoma in Northern Botswana, villages that span the urban-rural continuum. We discuss the challenges facing greywater management in such environments and review current management recommendations.

## 2. Experimental Section

### 2.1. Study Site

**Figure 1:**
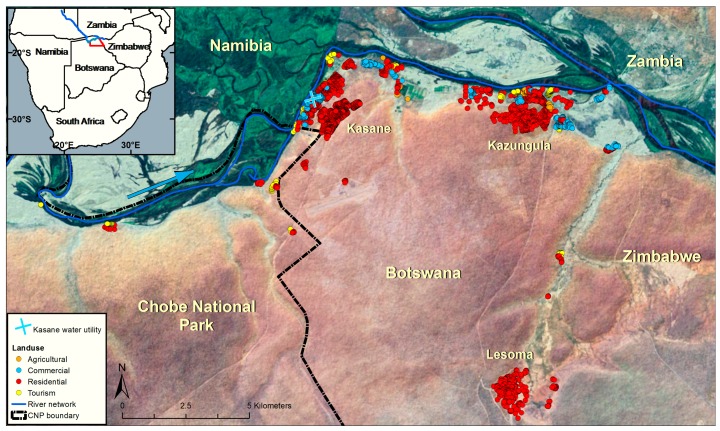
The study was conducted in Northern Botswana in nonsewered residential areas in the towns of Kasane, Kazungula, and Lesoma. Botswana is located in Southern Africa (inset). Buildings are noted by land use type (agricultural, commercial, residential and tourism associated). Residential dwellings are noted in red. Kasane and Kazungula are located near the Chobe River (blue line between Namibia and Botswana).

Botswana is a politically stable, semi-arid, landlocked country located in sub-Saharan Africa. The study was conducted in Chobe District (Figure 1). The district population (23,347 people) is spread across one urban community and eight smaller peri-urban and rural villages [22]. The country has a subtropical climate. The Chobe River, the source of all municipal water in the district, floods annually [23]. Piped water from local water treatment plants is available either through direct reticulation to residences or through access to public taps.

We surveyed 30 households in three non-sewered, low-income residential areas across three towns in Chobe District in 2013 (Figure 1): Kasane (pop. est. 9008), Kazungula (pop. est. 4113), and Lesoma (pop. est. 613, 2011 Botswana Population Census [22]). In order to compare density of dwellings by village, we estimated the number of dwellings per hectare in survey areas using the spatial analyst in ESRI ArcGIS (Version 10.2, Redlands, CA, USA) with buildings digitized using Google Earth imagery in 2010 [24], verified against field observations. 

### 2.2. Household Surveys

Thirty households were randomly selected in three different communities in Kasane, Kazungula and Lesoma. Data on pit latrine presence were determined through observation by the research team. Information on property ownership, household demographics, sanitation, water access, and greywater disposal methods were obtained through the use of questionnaires prepared in English (National languages are English and Tswana) and administered to the head of the household or oldest adult on the premises. The same member of the research team delivered the questionnaire with any Tswana translations provided by another member of team throughout the survey. All human associated data were anonymized. The research was conducted under permit from the Ministry of Health in Botswana and approval from the Virginia Tech Institutional Review Board (IRB# 11-573).

### 2.3. Statistical Analysis

All statistical analyses were conducted using the open source integrated programming environment R (R Core Team, 2013). Exact binomial confidence limits were calculated using the “binom” package. A chi-square test of goodness-of-fit was performed to evaluate equality of proportions with the “stats” package.

## 3. Results and Discussion

In dense residential environments where sanitation infrastructure is absent or limited, greywater can develop into an important public health threat. In our study, the majority of surveyed households were dependent on self-built, unsealed traditional pit latrines (69%, *n* = 90; 95% CI, 59%–79%), although some did report a complete lack of onsite sanitation (14%, 95% CI 0%–22%). Pit latrines were not emptied using vacuum-waste collection vehicles (honey sucker) as these were reported to be unavailable from local government and a major area of concern for residents. This was particularly so for those living in the Kasane survey area where a large number of respondents reported their onsite pit latrine was either full or almost full (13% and 46%, respectively, *n* = 24). An alternative approach for emptying pit latrines was observed at two properties and involved people manually removing excreta through a hole in the pit latrine wall created below ground level into an excavated hole adjacent to the structure. The hole in the pit latrine wall was then closed and the excreta in the adjacent hole covered with soil. Respondents indicated that this practice allowed continued use of the existing pit latrine structure without the financial investment needed to construct a new pit latrine. This method entails additional disease exposure risk to compound residents and, in particular, individuals undertaking the task.

### 3.1. Greywater Disposal Practices

**Figure 2:**
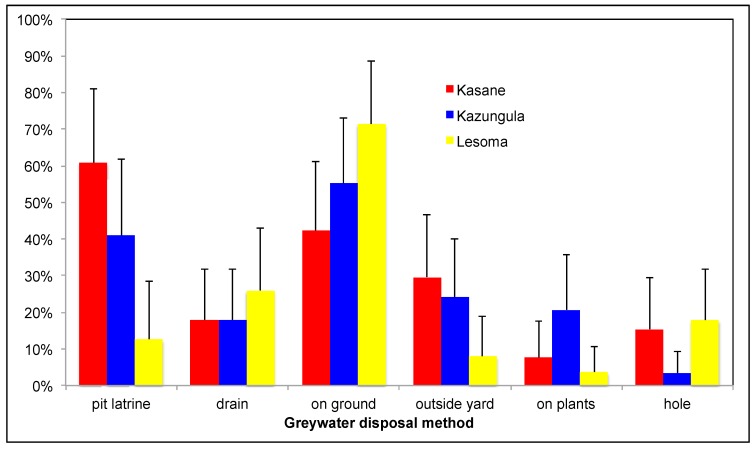
Greywater disposal varied by villages but dumping of greywater into the pit latrine was a practice found in all villages, particularly Kasane, where residential density was highest. While not statistically significant, there was a reverse trend for ground disposal of greywater to be higher in villages with lower density such as Kazungula and Lesoma.

Greywater waste disposal methods included dumping greywater directly on the ground within the compound, throwing it on plants in the compound, throwing it over the fence, dumping it into a hole also used for rubbish, pouring it down a drain connected to a septic tank, or dumping it into the pit latrine (Figure 2). Dumping of greywater into the pit latrine was a behavior identified in all survey areas. The proportion of households varied significantly across villages with Kasane having the highest frequency (61.9% (*p* < 0.014, χ^2^ = 9.13)) together with the highest density of dwellings per hectare (256 dwellings (du)/hectare, compare—Kazungula 100 du/ha and Lesoma 99 du/ha). While not statistically significant, there was a reverse trend for respondents to report greater ground disposal of greywater in villages with lower density such as Kazungula and Lesoma. Respondents indicated that the practice limited greywater contamination of the household compound with potentially infectious organisms and reduced odors. Owners also reported that dumping greywater into the pit latrine caused the level of fecal waste in the pit latrine hole to decrease, presumably through drainage of water and human waste through the unsealed soil bottom. Respondents reported a general lack of space and negative environmental impacts as driving factors influencing the decision to dispose of greywater in the pit latrine rather than on the ground around dwellings in the compound. In both Kasane and Kazungula, households reported that government officials had also instructed them to dump greywater into the pit latrine in order to avoid contamination of the household compound and health associated impacts. Reuse in surveyed households was limited and included application on plants including one respondent reporting that they used the greywater on their crops (Figure 2) and another in their brick building business undertaken at the residence.

### 3.2. Greywater and Hydraulic Loading of the Pit Latrine—Environmental Impacts

The presence of pit latrines has previously been associated with declines in water quality through bacterial contamination and nitrate leaching into water resources [25,26,27]. While rainfall and flooding events may influence pit latrine hydraulic loading and lead to contamination of groundwater resources under normal circumstances, habitual dumping of large quantities of greywater from other uses (bathing, washing, *etc.*) can dramatically transform the environmental footprint of the pit latrine. All pit latrines have a pit with walls, but the bottom of the pit is left unlined, requiring that it be constructed well above the groundwater table [28,29]. In contrast, septic tanks are enclosed sanitation structures that are very resistant to seepage from the tank [28]. They are designed to contain all greywater in addition to excreta and allow for a measure of treatment through biodegradation before the liquids are discharged into drain fields. Pit latrines, however, are not as robust as septic tanks in being able to withstand significant hydraulic loadings and have a substantially smaller holding volume available, usually designed for dry or near-dry loadings [28,29]. Even dual-pit pour flush latrines are not typically designed to withstand high hydraulic loadings.

With pit latrines, liquids can permeate the underlying soil, and, in cases where the ground is fractured rock as it is in residential areas surveyed in Kasane, there can be short-circuiting of liquid wastes to the groundwater table. The lack of a bottom lining or other similar type of containment structure in pit latrines makes them much more susceptible to bacterial transport out of the pit latrine and into surrounding groundwater with migration through that milieu to surface water or to groundwater wells. From there the exposure pathways can be many and direct.

Saturated soils can facilitate microorganism movement up to several hundred meters through subsurface water flow [30,31,32]. While most soil can effectively filter protozoa and helminthes, this is not the case for bacteria and viruses [33,34]. These microorganisms are typically transported through the subsurface along with the groundwater [34]. In areas with high groundwater tables, such as found near flatlands adjacent to rivers (as in parts of Kasane and Kazungula), transport of pathogens through the unsaturated soil zone to the saturated groundwater zone can be rapid, especially under certain hydraulic loading conditions. In these areas, the unsaturated zone is shallow and the distance from the point of soil entry to the saturated zone is short, thus providing for rapid movement from the unsaturated to the saturated zone.

Across interviewed household in the three villages, the greater majority of residential plots were used for rental purposes, either entirely or in part (80%). Across study areas we found very high household to pit latrine ratios (4.2 households, SD = 3.32, range = 15 units, average household size 5.3, SD = 4.4). Using the lower limit of the estimated greywater generation rate (15–55 L/capita/day [19]) and our mean household number and size, this equates to 334 L or more that could potentially be dumped into the pit latrine on a daily bases. This level of greywater disposal can significantly alter hydraulic loading, increasing the potential for leaching of microorganisms and parasites from the pit latrine environment. In this, hydraulic loadings may approach or exceed that of a septic tank without the benefits of the containment and fermentation features found in septic systems. The threat of microorganism movement under these conditions becomes elevated, presenting a significant risk to soil and ground and surface water resources. Bacterial migration through groundwater has been observed and studied for decades and presents a real risk in these systems (see, for example, [33,34]). Many factors play a role, however, in the survival and movement of pathogens in soil, including soil moisture content (higher survival rate in wet soil), moisture holding capacity (less sandy soils retain water better), temperature, pH, sunlight (greater survival under cloudy conditions), and others [34]. When the water table is high, leakage from pit latrines can travel far with viable pathogens potentially reaching drinking water sources, whether surface (rivers, lakes) or underground (wells).

At the household level, dumping of greywater into pit latrines removes the hazards of the waste from the compound and, according to respondents, reduces smells and the level of excrement in the pit latrine. This provides a positive feedback for this health behavior, a practice that appears to be replicated where space limitations may not be as significant (Kazungula and Lesoma) but positive impacts may encourage the behavior (e.g., reduction in fecal levels and odors). Indeed many respondents characterized dumping of greywater with detergents and soaps or pouring chemicals directly into the pit latrine (see below) as “cleaning” the pit latrine.

Pathogens are not the only concern for pit latrines that receive greywater but also chemical constituents that arise from the greywater itself or is dumped directly into the pit latrine [35]. For example, respondents reported dumping purchased chemicals directly into the pit latrine to control odors, in particular the disinfectant Jeyes Fluid (Jeyes Group Ltd., Norfolk, UK). This product contains chemical constituents that are toxic to humans and to aquatic organisms with the potential to bioaccumulate and cause long-term adverse effects in aquatic environments [36]. Some chemicals are also naturally present in human excrement (e.g., unmetabolized pharmaceutically active compounds) and can be mobilized and travel into groundwater aquifers [37,38]. A study in Nigeria identified contaminate impacts from pit latrines in water wells located 6–18 m away in the absence of observations of hydraulic overloading or direct loading of the pit latrine with chemicals.

### 3.3. Recommendations for Greywater Disposal

While important work has been done on greywater recycling options, it has been recommended that greywater management should focus on disposal only when onsite waterborne sanitation is absent [8]. Recommendations for greywater disposal have been developed from extensive work conducted in South Africa by Carden and colleagues [17] and provides important guidelines for management of greywater (Table 1 and Table 2). Following these recommendations, all three-village areas surveyed in this study should be provided with off-site greywater removal access. Given proximity to the river in Kasane and Kazungula, this is urgently required. While sewage systems may be developed in a particular area (e.g., under development in Kasane), poverty may still influence the use of such structures given the inability of many to pay for connections and necessary infrastructure. In congested areas where poverty is persistent, development of public drain access to existing sewage systems should be prioritized for greywater disposal. While households are willing to travel to collect water, they will not often do the same for disposal [17], requiring public access to be convenient if it is going to be effective. Other alternatives should be explored including options for greywater recycling, particularly in the dryland countries such as Botswana where water demand management is an increasing challenge [39,40].

**Table 1:** Factors Influencing Greywater Management Approaches in non-sewered areas (adapted from Carden *et al.* [17]).

1. Water use and greywater disposal behavior;
2. Water consumption (Off site removal recommended when greywater generation rate >2500 L/ha per day);
3. Human settlement density (off-site removal is recommended when density >50 du/ha);
4. Soil surface properties, drainage, and previous disposal practices (e.g., build up of grease and scum); Off site removal is recommended when surfaces are hard packed and more impervious (clay and rock);
5. Topography and slope—potential for erosion and/or ponding of greywater; off site removal recommended when the slope of the area is greater than 30%;
6. Rainfall—potential for surface flow of polluted waters to low-lying areas or decreased drainage due to water logged soils;
7. Depth to water table—where the water table is high, soak away systems may not be able to absorb greywater and the risk of pollution of ground water is elevated;
8. Proximity to sensitive environments—wastewater pollution of rivers, wetlands, unprotected boreholes, or floodplains;
9. Current wastewater management methods—existing initiatives and infrastructure should be considered in devising disposal strategies.

Guidelines provide an important reference for strategic planning but interventions must be developed in respect of the local environment. Demand responsive, community based, or household centered approaches are recommended when working towards water, sanitation, and health (WASH) goals in developing countries [41]. Interventions that are not reflective of the local situation will likely have poor uptake and will fail to address emergent issues facing the community in question—there is no “one size fits all” in development. The central element of these approaches is the requirement to (1) respond directly to needs and demands of the target group; (2) ensure that the household and associated neighborhoods are at the center of the planning process; and (3) stakeholder participation is identified and includes all relevant actors [8]. 

**Table 2:** Greywater management guidelines from Carden *et al.* [17]. Environmental impacts of greywater disposal practices will be influenced by important environmental factors including soil surface properties, topography, water table depth, and proximity of the area to environmentally sensitive environments (see Table 1).

Settlement Density	Greywater Generation Rate (ℓ/day)	Density (du/ha)	Plot Size (m^2^)	Greywater Disposal Option
Low	<500	<10	>800	Soakaways at water collection points and stand pipes.
Low/Medium	500–1500	10–30	300–800	Soakaways must be installed at standpipes, if water is reticulated to the dwelling (yard or home) recommend connection to on- or off-site disposal system.
Medium/high	1500–2500	30–50	150–300	If water is reticulated to the dwelling (yard or home) connection to on- or off-site disposal system must be installed, formal washing areas must also be developed with appropriate disposal systems.
High	>2500	>50	<150	Off-site disposal

## 4. Conclusions

Human sanitation and hygiene behavior can have complex effects on the environment, domestic and wild animals, and public health. While traditional pit latrines address the need for disposal of human excreta, wastewater persists as an unaddressed environmental health problem in many congested residential areas. In such communities, it is necessary to ensure that wastewater and hazardous chemicals are not being dumped into latrines. Greywater disposal patterns and pit latrine management practices should be evaluated and monitored such that hazardous health behaviors and emergent negative environmental conditions can be identified and managed early, particularly in congested residential areas where pit latrines are the dominant form of sanitation. Management and intervention strategies should be developed directly with communities and other stakeholders. These efforts can contribute to the development of broader guidelines for greywater disposal that is integrated into national WASH objectives, procedures, and service delivery. WASH professionals should be trained in an integrated manner, ensuring that community directives are harmonious with overall environmental health objectives across sectors.

## References

[1] Carson R. (2002). Silent Spring.

[2] Cohen B. (2006). Urbanization in developing countries: Current trends, future projections, and key challenges for sustainability. Technol. Soc..

[3] Sclar E.D., Garau P., Carolini G. (2005). The 21st century health challenge of slums and cities. Lancet.

[4] Imhof B., Muhlemann J. (2005). Greywater Treatment on Household Level in Developing Countries—A State of the Art Review.

[5] Katukiza A., Ronteltap M., Niwagaba C., Kansiime F., Lens P. (2015). Grey water characterisation and pollutant loads in an urban slum. Int. J. Environ. Sci. Technol..

[6] Ghaitidak D.M., Yadav K.D. (2013). Characteristics and treatment of greywater—A review. Environ. Sci. Pollut. Res..

[7] Eriksson E., Auffarth K., Henze M., Ledin A. (2002). Characteristics of grey wastewater. Urban Water.

[8] Winter K., Spiegel A., Armitage N., Carden K. (2011). Sustainable Options for Community Level Management of Greywater in Settlements without On-Site Waterborne Sanitation.

[9] Carden K., Armitage N., Winter K., Sichone O., Rivett U., Kahonde J. (2007). The use and disposal of greywater in the non-sewered areas of South Africa: Part 1—Quantifying the greywater generated and assessing its quality. Water SA.

[10] Carden K., Armitage N., Winter K., Sichone O., Rivett U. (2007). Understanding the Use and Disposal of Greywater in the Non-Sewered Areas of South Africa.

[11] Ridderstolpe P. (2004). Introduction to Greywater Management.

[12] Ottoson J., Stenström T.A. (2003). Faecal contamination of greywater and associated microbial risks. Water Res..

[13] Birks R., Hills S. (2007). Characterisation of indicator organisms and pathogens in domestic greywater for recycling. Environ. Monit. Assess..

[14] Rose J.B., Sun G.-S., Gerba C.P., Sinclair N.A. (1991). Microbial quality and persistence of enteric pathogens in graywater from various household sources. Water Res..

[15] Govender T., Barnes J.M., Pieper C.H. (2011). Contribution of water pollution from inadequate sanitation and housing quality to diarrheal disease in low-cost housing settlements of cape town, South Africa. Am. J. Public Health.

[16] Carden K., Armitage N., Winter K., Sichone O., Rivett U. (2008). The management of greywater in the non-sewered areas of South Africa. Urban Water J..

[17] Carden K., Armitage N., Sichone O., Winter K. (2007). The use and disposal of greywater in the non-sewered areas of South Africa: Part 2—Greywater management options. Water SA.

[18] Graham N. (2003). A review of Infrastructure Services for the Upgrading of South African Informal Settlements. Master’s Thesis.

[19] Mekonnen M.M., Hoekstra A.Y. (2011). National Water Footprint Accounts: The Green, Blue and Grey Water Footprint of Production and Consumption.

[20] Jobbins S.E., Alexander K.A. (2015). From whence they came—Antibiotic-resistant *Escherichia coli* in African wildlife. J. Wildl. Dis..

[21] Pesapane R., Ponder M., Alexander K. (2013). Tracking pathogen transmission at the human-wildlife interface: Banded mongoose and *Escherichia coli*. Ecohealth.

[22] Botswana Government, Central Statistics Office (2011). 2011 Botswana Population and Housing Census Alphabetical Index of Villages.

[23] Gaughan A., Waylen P. (2012). Spatial and temporal precipitation variability in the Okavango‚ Kwando‚ Zambezi catchment, southern Africa. J. Arid Environ..

[24] Google Earth. https://www.google.com/earth/.

[25] Jacks G., Sefe F., Carling M., Hammar M., Letsamao P. (1999). Tentative nitrogen budget for pit latrines‚ äìeastern Botswana. Environ.Geol..

[26] Still D., Nash S. (2002). Groundwater Contamination due to Pit Latrines Located in a Sandy Aquifer: A Case Study from Maputaland.

[27] Ahaneku I.E., Adeoye P.A. (2014). Impact of pit latrines on groundwater quality of Fokoslum, Ibadan, southwestern Nigeria. Brit. J. Appl. Sci. Technol..

[28] Frieden T.R., Damon I., Bell B.P., Kenyon T., Nichol S. (2014). Ebola 2014—New challenges, new global response and responsibility. N. Engl.J. Med..

[29] Anyamba A., Chretien J.-P., Small J., Tucker C.J., Formenty P.B., Richardson J.H., Britch S.C., Schnabel D.C., Erickson R.L., Linthicum K.J. (2009). Prediction of a rift valley fever outbreak. Proc. Natl. Acad. Sci. USA.

[30] Nichols D.S., Prettyman D., Gross M. (1983). Movement of bacteria and nutrients from pit latrines in the boundary waters canoe area wilderness. Water Air Soil Pollut..

[31] Stiles C., Crohurst H. (1923). The principles underlying the movement of *Bacillus coli* in ground water with resultant pollution of wells. Public Health Rep..

[32] McCoy E., Rahe T., Kling G., Hagedorn C. (1978). Transport of antibiotic-resistant *Escherichia coli* through western oregon hillslope soils under conditions of saturated flow. J. Environ. Qual..

[33] Hagedorn C., Hansen D.T., Simonsson G.H. (1978). Survival and movement of fecal indicator bacteria in soil under conditions of saturated flow. J. Environ. Qual..

[34] Gerba C.P. (1975). Fate of waste water bacteria and viruses in soil. J. Irrig. Drain. Div..

[35] Almqvist H., Hanæus J. (2006). Organic hazardous substances in graywater from Swedish households. J. Environ. Eng..

[36] Jeyes Fluid, Safety Data Sheet. http://dchd.co.uk/admin/SDS_JeyesFluid1L.pdf.

[37] Carballa M., Omil F., Lema J.M., Llompart M.A., García-Jares C., Rodríguez I., Gomez M., Ternes T. (2004). Behavior of pharmaceuticals, cosmetics and hormones in a sewage treatment plant. Water Res..

[38] Heberer T. (2002). Tracking persistent pharmaceutical residues from municipal sewage to drinking water. J. Hydrol..

[39] Esrey S.A. (2001). Towards a recycling society: Ecological sanitation-closing the loop to food security. Water Sci. Technol..

[40] Madungwe E., Sakuringwa S. (2007). Greywater reuse: A strategy for water demand management in harare?. Phys. Chem. Earth Parts A/B/C.

[41] Sigel K., Altantuul K., Basandorj D. (2012). Household needs and demand for improved water supply and sanitation in peri-urban ger areas: The case of Darkhan, Mongolia. Environ. Earth Sci..

